# Supplementary material for: Heterogeneous networks: Fair power allocation in LTE-A uplink scenarios
Source: PLoS One. 2021 Jun 21;16(6):e0252421. doi: 10.1371/journal.pone.0252421 (PMC8216560; doi:10.1371/journal.pone.0252421)
Supplement: Attachment — Submitted filename: PONE Reviewers Comments.docx [file pone.0252421.s001.docx]

Answer to the Reviewer’s comments and description of the changes introduced in the revised version of the submitted manuscript

# Reviewer #1:

(1) The manuscript need thorough copyedit for language usage, spelling and grammar.

**Reply to the general remarks:** The authors appreciate the overall evaluation of the paper, as well as the thorough review and valuable suggestions.

The manuscript has been carefully revised by an expert. Please, see some examples below.

**Abstract:**

**First paragraph in section 1**

**Second paragraph in section 1**

**Third paragraph in section 1**

**Fourth paragraph in section 1**

**Last paragraph in section 1**

**First and second paragraphs in section 2**

**6th paragraphs in section 2**

**Section 4.2**

**Section 5**

**Section 6**

**Section 7**

(2) The terminology of the contribution is unclear - whether it is to promote new strategy, scheme, algorithm or method.

| Reviewer Comment | 2 |
| --- | --- |
| Authors’ response | The author agrees with the reviewer’s comment. The terminology must be unified. Since this study proposed a new strategy, the other terms were removed according to the reviewer’s comment. |

(3) Contribution in Section 3 should be revised to make it specific, measurable and related to the results. For example there are no results presented to show that it has low computational complexity and high precision.

| Reviewer Comment | 3 |
| --- | --- |
| Authors’ response | In order not to let the readers into error, the title of the section and its content have been revised. According to the reviewer’s comment, the non-specific contexts have been deleted, and the others have been altered. |

# Reviewer #2:

This paper covers a timely and interesting topic. This reviewer has the below comments:

1. The authors should provide a table summarizing the main notation that has been adopted in the manuscript.

The authors appreciate the reviewer’s comprehensive comments, which further improved the quality of the paper.

The author did not consider the notation due to the fact that it has not been required in the b Submission Guidelines.

2. Section 3 and 7 cannot be count as the section which are very short. Marge these sections with other sections.

The author thanks the reviewer for all his/her concerns. The author agrees with the reviewer that section 3 is not long enough to have a section, but it is related to the most important part of the paper which is the research significance.

Regarding section 7, based on the Submission Guidelines, the conclusion section must be in an individual section.

3. References are not up to date. Need to align with updated literature

The References have been updated up to 13/02/2021 according to the reviewer’s comment.

# Reviewer #3:

The subject is considered as not new as most of the references made were published more than 5 years. Since the current research trend is moving towards beyond 5G, the topic of LTE-A which is considered as 4G is considered as has been studied well. Moreover the manuscript needs to be proof read since many grammatical errors could be found.

The authors appreciate the overall evaluation of the paper, as well as the thorough review and valuable suggestions.

1. According the reviewer’s remark the references were updated.
2. The proposed strategy is not only related to the LTE-A or 5G. In fact, the main idea of this work can be used for all generation devices. The author tried to propose a novel strategy to solve a specific issue explained in section 1. Simply, the author tried to mitigate the interference in heterogynous networks. In other words, method can be considered an applicable and new strategy because it overcomes the interference issues in two respective conditions (i) targeting minimum threshold for the RT services and (ii) then applying boosting factor that enables adapting the power allocation proportionally to the femtocells/Macrocell' role in the interference situation rather than uniformly as usually performed in traditional power adjustment approaches. Thus, the proposed strategy can be easily used by any mobile developer companies.
3. The manuscript has been carefully revised by an expert. Please, see some examples above given in the first comment – Reviewer 1.

6. PLOS authors have the option to publish the peer review history of their article ([what does this mean?](https://journals.plos.org/plosone/s/editorial-and-peer-review-process#loc-peer-review-history)). If published, this will include your full peer review and any attached files.
If you choose “no”, your identity will remain anonymous but your review may still be made public.
**Do you want your identity to be public for this peer review?** For information about this choice, including consent withdrawal, please see our [Privacy Policy](https://www.plos.org/privacy-policy).

Reviewer #1: No

Reviewer #2: **Yes:** Dr. Ferdous Hossain

Reviewer #3: No

[NOTE: If reviewer comments were submitted as an attachment file, they will be attached to this email and accessible via the submission site. Please log into your account, locate the manuscript record, and check for the action link "View Attachments". If this link does not appear, there are no attachment files.]
For journal use only: PONEDEC3

1. Is the manuscript technically sound, and do the data support the conclusions?
The manuscript must describe a technically sound piece of scientific research with data that supports the conclusions. Experiments must have been conducted rigorously, with appropriate controls, replication, and sample sizes. The conclusions must be drawn appropriately based on the data presented.

Reviewer #1: Partly

Reviewer #2: Partly

Reviewer #3: Partly

2. Has the statistical analysis been performed appropriately and rigorously?

Reviewer #1: Yes

Reviewer #2: Yes

Reviewer #3: No

3. Have the authors made all data underlying the findings in their manuscript fully available?
The [PLOS Data policy](http://www.plosone.org/static/policies.action#sharing) requires authors to make all data underlying the findings described in their manuscript fully available without restriction, with rare exception (please refer to the Data Availability Statement in the manuscript PDF file). The data should be provided as part of the manuscript or its supporting information, or deposited to a public repository. For example, in addition to summary statistics, the data points behind means, medians and variance measures should be available. If there are restrictions on publicly sharing data—e.g. participant privacy or use of data from a third party—those must be specified.

Reviewer #1: Yes

Reviewer #2: Yes

Reviewer #3: Yes

4. Is the manuscript presented in an intelligible fashion and written in standard English?
PLOS ONE does not copyedit accepted manuscripts, so the language in submitted articles must be clear, correct, and unambiguous. Any typographical or grammatical errors should be corrected at revision, so please note any specific errors here.

Reviewer #1: No

Reviewer #2: Yes

Reviewer #3: No

5. Review Comments to the Author

Please use the space provided to explain your answers to the questions above. You may also include additional comments for the author, including concerns about dual publication, research ethics, or publication ethics. (Please upload your review as an attachment if it exceeds 20,000 characters)
