# Supplementary material for: Heterogeneous networks: Fair power allocation in LTE-A uplink scenarios
Source: PLoS One. 2021 Jun 21;16(6):e0252421. doi: 10.1371/journal.pone.0252421 (PMC8216560; doi:10.1371/journal.pone.0252421)
Supplement: Attachment — Submitted filename: Reviewer comments I_1619804577.docx [file pone.0252421.s002.docx]

**Answer to the Reviewer’s comments**

**Editor's comments:**

Change the referencing style from numbers to authors’ surnames followed by the publication year.

**Reply:** Done as requested.

……………………………………………………………………………………………………………………………………………

# Reviewer #1:

The authors study the problem off interference mitigation within long term evolution networks by providing initially a review analysis of the existing works that studied the impact of power control techniques in the interference mitigation regarding the network performance, and then the authors introduce an interference mitigation model in order to improve the operation of the LTE networks focusing on the uplink communication.

The authors have provided a detailed review of some existing methods studying the power control problem in the uplink of LTE networks and they have tried to categorize those approaches. Then, they introduce a femtocell-based architecture where they deal with a power control problem aiming at the interference mitigation.

They have introduced an algorithmic approach in order to deal with the power control problem and they examined several topologies in order to evaluate the proposed framework in terms of achievable throughput and the corresponding signal to interference plus noise ratio. The authors should consider the following suggestions provided by the reviewer in order to improve the quality of presentation of their manuscript, as well as its scientific depth.

Reply to the general remarks: The authors appreciate the overall evaluation of the paper, as well as the thorough review and valuable suggestions.

First comment:

(A) Initially, in Section 2, the authors should discuss the existing resource management approaches, such as Tsiropoulou, E. E., et al. "Uplink resource allocation in SC-FDMA wireless networks: A survey and taxonomy." Computer Networks 96 (2016): 1-28, exploiting the single carrier frequency division multiple access technique in long-term evolution networks in order to deal with the problem of interference mitigation and power control.

(B) Furthermore, the problem of power control and interference mitigation is jointly examined with the problem of bandwidth allocation, such as Tsiropoulou, E. E., et al. "Energy-efficient subcarrier allocation in SC-FDMA wireless networks based on multilateral model of bargaining." 2013 IFIP Networking Conference. IEEE, 2013, Hyung G. Myung, Junsung Lim, David J. Goodman, “Single carrier FDMA for uplink wireless transmission,”IEEE Vehicular Technology Magazine, Vol. 1, Issue 3, pp. 30 – 38, Feb. 2007, in order to provide realistic and holistic solutions that exploit the available resources of the network .

The authors should improve the provided survey study in Section 2 in order to identify the existing approaches and better clarify their main contributions.

| Reviewer Comment | 1 (A and B) |
| --- | --- |
| Authors’ response | The author agrees with the reviewer’s remark. Further details regarding the existing resource management approaches must be shown in the manuscript.  According to the reviewer’s comment, the author has altered sections 1 and 2. Accordingly, the mentioned references have been added to the section. |
| Notes/action | Please, see 7^th^ paragraph in section 2  **Moreover, Tsiropoulou et al. [34] made an extensive survey to resource allocation algorithms in multiuser SC-FDMA wireless network through throughput optimization, resource allocation and fairness, resource allocation considering user’s Qos requirements, and joint power and subcarrier allocation. Accordingly, the authors reported several promising paths to follow heterogeneity network design and implement a resource allocation scheme such as subcarriers/RBs and users’ uplink transmission power with respect to multiple services (VoIP and video flow) which is related to the scope and novelty of this study**.  Please, see 2^nd^ paragraph in section 1  The battery life can be considered as one of the key parameters that significantly affect the efficiency of all mobiles. Although battery performance has been improved by many manufactures, it is still necessary to ensure that the mobile uses as low battery power as possible. As mentioned above, LTE-A signal format has been using SC-OFDMA to provide reliable high speed data communications. **Similar fact has been reported by Hyung et al. [1]**. The use of OFDM has enabled LTE to provide reliable link quality even in the presence of reflections and the adaptive modulation which provides the ability to modify the link according to the prevailing signal quality.  Please, see 6^th^ paragraph in section 2  To optimize the problem of energy efficiency in the resources allocation in a heterogeneous network scenario, Zhang et al. [30] proposed a new power and sub-channel algorithm allocations according to the number of the users and their demands. Accordingly, they proposed to use classical small/macro cell case networks instead of the conventional systems. Similar concept can be seen in the study of Chen et al. [31] regarding the optimization of the UL–DL decoupling use, and Iturralde et al. [32] for radio resource management schemes. **Furthermore, Tsiropoulou et al. [33] focused on the optimum range of power allocation and subcarrier in multiuser SC-FDMA wireless networks through the tradeoff between users’ actual uplink throughput performance and their corresponding energy consumption. Nevertheless, this work did not focus on heterogeneous network.**  **New references**   1. Myung, H. G., et al. (2006). "Single carrier FDMA for uplink wireless transmission." IEEE Vehicular Technology Magazine 1(3): 30-38. 2. Tsiropoulou, E. E., et al. (2013). Energy-efficient subcarrier allocation in SC-FDMA wireless networks based on multilateral model of bargaining. 2013 IFIP Networking Conference, IEEE. 3. Tsiropoulou, E. E., et al. (2016). "Uplink resource allocation in SC-FDMA wireless networks: A survey and taxonomy." Computer Networks 96: 1-28. |

2. In Section 3, the authors seem to adopt a code division multiple access technique, which is relatively outdated in long term evolution networks. The authors should better clarify the choice of multiple access technique in order to perform their analysis.

| Reviewer Comment | 2 |
| --- | --- |
| Authors’ response | The author of this study also agrees that adopt a code division multiple access technique is outdated. In fact, this study considered SC-OFDMA for the UL.  In order not to lead the readers into error, a statement was added to the mentioned section. |
| Notes/action | Please, see section 3 in the new manuscript  4. **Since the SC-OFDMA technology is mainly introduced to transfer data in LTE-A UL direction, the proposed strategy simulated the RT services by the same technology (SC-OFDMA) with a scenario that considers VoIP flows and video.**  5. This method can be considered an applicable strategy because it overcomes the interference issues in two respective conditions (i) targeting minimum threshold for the RT services and (ii) then applying boosting factor that enables adapting the power allocation proportionally to the femtocells/Macrocell' role in the interference situation rather than uniformly as usually performed in traditional power adjustment approaches. Thus, the proposed strategy can be easily used by any mobile developer companies. |

3. In section 6, the authors should include some additional numerical results for a large scale set up with a large number of connected nodes in order to show the scalability, efficiency, and robustness of the proposed framework. This discussion should be complemented with some additional numerical simulations quantifying the computational complexity of the proposed framework.

| Reviewer Comment | 3 |
| --- | --- |
| Authors’ response | In order to confirm that the scalability, efficiency, and robustness of the proposed framework a large scale set up with a large number of connected nodes was considered. Moreover each simulation scenario from 1-10 femtocell was repeated 30 times. At each time, the Macrocell user was positioned in different places in order to get a data more realistic. Accordingly, this feature implemented by using srand() function. Then, the average data was considered to get the performance of Femtocell users in terms of throughput. In addition, the time flow at each scenario specified by 300s which gave the gigabyte to data flow to both services VoIP and Video.  According to the reviewer’s suggestion, the above statement was added to the manuscript. |
| Notes/action | Please, see the 5^th^ paragraph in section 6.1 in the new manuscript  In order to confirm that the scalability, efficiency, and robustness of the proposed framework a large scale set up with a large number of connected nodes was considered. Moreover each simulation scenario from 1-10 femtocell was repeated 30 times. At each time, the Macrocell user was positioned in different places in order to get a data more realistic. Accordingly, this feature implemented by using srand() function. Then, the average data was considered to get the performance of Femtocell users in terms of throughput. In addition, the time flow at each scenario specified by 300s which was gave the GiG to data flow to both services VoIP and Video. |

4. Finally, the overall manuscript should be checked for typos, syntax, and grammar errors in order to improve the quality of its presentation.

| Reviewer Comment | 4 |
| --- | --- |
| Authors’ response | The author asked an expert to revise the manuscript in terms of typos, syntax, and grammar errors |

Finally, the author of this study believes that all comments of the reviewer have been carefully considered. Due to these valuable comments, the quality of the manuscript is now significantly improved. The author of this study will gladly accept any other comments from the reviewer. Once again, thank you very much for your significant efforts and valuable time.

# Reviewer #2

In general, authors perform a detailed analysis on the targeted topic. The work looks solid. However, I have some comments that authors may concern:

The authors appreciates the reviewer’s comprehensive comments, which further improved the quality of the paper.

1) the paper is entitled by "Heterogeneous Networks: Fair Power Utilization in LTE-A Uplink scenarios". However, in your manuscript, power utilization is not mentioned insufficiently. For example, in fig 2, only section mentions general power problems.

| Reviewer Comment | 1 |
| --- | --- |
| Authors’ response | According to the author’s experience, the terms power utilization, power allocation and power consumption are all related to the domain of this study which is about minimize the amount of power that has transmitted from each direction (Uplink and Downlink)  Nevertheless, the author of this study agrees with the reviewer’s remark. The term power allocation might be more adequate to the title of the study. |
| Notes/action | Please, see the title of the new manuscript.  **Old title:**  Heterogeneous Networks: Fair Power UTILIZATION in LTE-A Uplink Scenarios  **New title:**  Heterogeneous Networks: Fair Power **ALLOCATION** in LTE-A Uplink Scenarios |

2) I suggest authors may further provide the concepts of Generation Partnership Project. In my opinion, traditional multi-layer networks cannot be regarded as Uplink (UL) direction. Authors should clarify these concepts.

| Reviewer Comment | 2 |
| --- | --- |
| Authors’ response | According to the LTE-A specification (3GPP Release NO. 11) made by Rohde and Schwarz which is entitled “LTE- Advanced (3GPP Rel.11) Technology Introduction - White Paper”, specifically section 2.2, section 2.2.2 (Downlink CoMP), section 2.2.3 (Uplink CoMP), coordination multi-point operation has standardized for the Uplink HetNets based on the interference scenario for both resources scheduling and Beamforming (Power allocation).  The above argument can be clearly seen in the mentioned specification (3GPP Release NO. 11) as shown below (see page 15).  *For future HetNet deployment scenarios, where a macro cell provides the coverage and several small cells are used for capacity, there is higher uplink interference at the cell boundaries. This is especially true for the case, that macro cell and small cells are using the same cell identities. You can find the paper at*  3GPP Release NO. 11:  [*https://cdn.rohde-schwarz.com/pws/dl_downloads/dl_application/application_notes/1ma232/1MA232_1e_LTE_Rel11_technology.pdf*](https://cdn.rohde-schwarz.com/pws/dl_downloads/dl_application/application_notes/1ma232/1MA232_1e_LTE_Rel11_technology.pdf)  Based on the reviewer’s recommendation, the authors of this study added further details regarding the matter in section 1 in order not to let the readers into error. |
| Notes/action | Please, paragraph NO. 7 in section 1.  **According to the LTE-A specification (3GPP Release NO. 11) made by Rohde and Schwarz (Schwarz and Rohde 2014) which is entitled “LTE- Advanced (3GPP Rel.11) Technology Introduction - White Paper”, the coordination multi-point operation (CoMP) has standardized for the Uplink HetNets based on the interference scenario for both resources scheduling and Beamforming (Power allocation). Moreover**, specific control techniques for the UE transmission power are addressed to minimize the negative effect of the interference on the network capacities. Furthermore, there were 2 mechanisms introduced for Resources Block (RB) TPC in the LTE uplink: (i) Open Loop Power Control Point (OLPC) and (ii) Dynamic Power Offset (DPO) that provide the basic operating point for UE transmit power (TP) as shown below: |

3) Authors may further mention the heterogeneous networks, such as done in zhang, et al. Nature-Inspired Self-Organization, Control, and Optimization in Heterogeneous Wireless Networks, ieee tmc, 2012. Authors may also investigate other types of such networks.

| Reviewer Comment | 3 |
| --- | --- |
| Authors’ response | The author agrees with the reviewer’s remark. Further details regarding the existing heterogeneous networks approaches must be shown in the manuscript.  According to the reviewer’s comment, the author added a new paragraph. Accordingly, the mentioned references have been cited in the manuscript. |
| Notes/action | Please, paragraph NO. 6 in section 2.  Bennis et al. [28] analyzed the influence of various eNobeB sizes on the PC, assuming femtocells' overhearing ability of MUEs' RSRP measurements. DL power allocation methods were proposed in some studies [29, 30], where no control information exchange was considered between HeNobeBs and eNobeBs. As an alternative, each HeNobeBs predicts the RB allocation to its close-by MUEs according to their overheard Channel State Information (CSI) feedback from MUEs to eNobeBs. According to the MUE allocation prediction, each HeNobeBs can perform resource allocation to FUEs while mitigating interference towards close-by MUEs. Nevertheless, interference mitigation (IM) among FBSs was not considered. **Milner et al. [31] proposed a new Flocking Algorithm for self-organization and control of the backbone nodes in the hierarchical heterogeneous wireless networks through collecting local information from end users. Additionally, the new algorithm suggested by the authors can sufficiently work for the energy optimization while respect to convergence speed and quality of solutions.** |

4) Tabulate the major findings from your previous analysis. There is no proper explanation under section 2.

| Reviewer Comment | 4 |
| --- | --- |
| Authors’ response | Thank you very much for your comment. In my previous works, specifically during my PhD and later as a post doc researcher, I proposed different models to mitigate interference in heterogeneous network which are all about adjusting power in the “Downlink direction”. While, the target of this study is related to the same subject (Power Allocation) but in the Uplink direction.  In addition, the problematic in the two directions (uplink and downlink) are different. In Downlink, we investigate to get higher performances to macro cell user equipment. The opposite is true for uplink direction. Since the domain of this study relates to the uplink direction, the author of this study investigated a model to get higher performance to small cell user equipment.  According to the reviewer’s comment, the author has explained the above matters and guided the readers to find the major findings from my previous analysis. |
| Notes/action | Please, paragraph NO. 11 in section 1.  **In general, the problematic of interference in uplink and downlink are different subjects. In Downlink direction, higher performances to macro cell user equipment will be the main target [9, 11, 19-23]. While, in uplink direction, higher performances to small cell user equipment will be the main target. Since the domain of this study relates to the uplink direction, the authors of this study investigated a model to get higher performance to small cell user equipment. Accordingly to overcome the issues mentioned points (i and ii)**, this study utilizes a novel strategy for the DPO scheme. In this strategy, the uplink receiver at HeNodeB estimates the SINR of the received signal and compares it with the desired SINR target value according to Real Time Traffics (RTT). Based on the mentioned parameters, this study named the strategy “Fractional Self-Power Control (FSPC)”. In the proposed strategy, TPC command is transmitted to UE to increase the power transmitter power when the received SINR does not satisfy the SINR target. Otherwise, the TPC command will be requested to decrease a transmitter power. In other words, the FSPC specifies the minimum transmission for each UE in the first step. Then, the initial power will be further adjusted using PDCCH signaling as a TPC commands to the UE. |

5) Author may clear the contributions with case study approach.

| Reviewer Comment | 5 |
| --- | --- |
| Authors’ response | In order not to let the readers into error, the comment of the reviewer has been considered and the sections that relate to contributions have been altered. |
| Notes/action | Please, see the first paragraph in section 1.  The innovations of this paper are summarized on the following aspects **(i-v). In other words, they can be considered as a main contribution of the study.**  The points (i-v) have been shown in section 3.  Please, see the first paragraph in section 6.1.  **For that purpose, two different scenarios which they are considered as the main contribution of this work have been implemented as follows:**   - First, the heterogeneous network deployment with femtocell deployed near the macro cell eNodeB was examined, named macrocell cell center area (see Figure 1). - Second, the heterogeneous network deployment with femtocells distributed from the macro cell eNodeB is examined, named macrocell cell edge area (see Figure 2). |

6) Results must be properly discussed under the discussion section. There is no result comparison or analysis identified in this paper.

| Reviewer Comment | 6 |
| --- | --- |
| Authors’ response | Based on the reviewer’s valuable remark, I have tried to find the most related studies and compare with the output of this work.  In addition, I also supported the explanation based on the previous studies. |
| Notes/action | Please, see the last paragraph in section 6.2.1  Accordingly, it’s evaluated; the performance gain of the proposed strategy is more than 3.5% and 1.2% of the Video and VoIP. **The above facts, specifically (the SINR versus the distance of MUEs as a function of impact interference in the HetNets ) has been also investigated by a study [31]. Nevertheless, the mentioned study has not focused on a real scenario such as the motilities of the UEs either eNodeB/HeNodeB, as well as there is no detail on the way how the UEs have applied to the selected service applications such as VoIP or Video. While the amount of power could be dynamically changed according to the MCS of each UE service. A study of Alsafasfeh et al. [72] worked on the efficient power control framework for small-cell heterogeneous networks, and developed an optimization framework that allows the small-cell base station to switch off when the traffic demands are not critical. However, this might not be a practical solution.**  Please, also see other parts of the new manuscript  As indicated the interference level reduces when applying the FSPC approach over the static Pmax. It is also considered that due to the absence interfering MUEs toward FUEs, the interference level given to the FUES cell center decreased [24-27, 31].  The figures also show that the average Pmax of the small cells are not excited 16.07 dBm equivalent to the same interference level as compared to the reference cases with Pmax of 23dBm in all femtocell cell deployments. This consideration guarantee that the received SINR is enhanced because of the dropped interference from each the interfering FUEs and MUEs in this deployment scenario [16, 71].  Figures 6 and 7 illustrate the packet loss ratio PLR of Video and VoIP flows respectively. It can be seen from each figure that the PLS increases when the number of HeNodeB increased. This is because the number of uploading FUEs is highly correlated to the generating interference [31] |

7) Add some new research and avoid repeated concepts in your manuscript.

| Reviewer Comment | 7 |
| --- | --- |
| Authors’ response | Done as requested.  See comments  1. Haider, A., R.S. Sinha, and S.H. Hwang, *Investigation of Open‐Loop Transmit Power Control Parameters for Homogeneous and Heterogeneous Small‐Cell Uplinks.* ETRI Journal, 2018. **40**(1): p. 51-60.  2. Li, J. *Uplink power control for heterogeneous networks*. in *2013 IEEE Wireless Communications and Networking Conference (WCNC)*. 2013. IEEE.  3. Vallejo-Mora, A.B., et al., *Congestion relief in subway areas by tuning uplink power control in LTE.* IEEE Transactions on Vehicular Technology, 2016. **66**(7): p. 6489-6497.  4. Cîrstea, E.-R. and S. Ciochină. *LTE Uplink Power Control and its Impact on System Performance*. in *Proceedings of 3rd International Conference on Adaptive and Self-Adaptive Systems and Applications*. 2011. Citeseer.  5. Koleva, P., et al. *Improved open loop power control for LTE uplink*. in *2015 38th International Conference on Telecommunications and Signal Processing (TSP)*. 2015. IEEE.  6. Kim, W., Z. Kaleem, and K. Chang, *Power headroom report-based uplink power control in 3GPP LTE-A HetNet. EURASIP J.* Wireless Com. Network, 2015. **233**: p. 2015.  7. Mohamed, K.S., M.Y. Alias, and M. Roslee, *Review on femto-cell networks interference management techniques.* International jouranl of engineering and technology, 2018. **10**(4): p. 1248-1262. |

Finally, the author of this study believes that all comments of the reviewer have been carefully considered. Due to these valuable comments, the quality of the manuscript is now significantly improved. The author of this study will gladly accept any other comments from the reviewer. Once again, thank you very much for your significant effort and valuable time.
